# Maternal Obesity in Pregnancy Developmentally Programs Adipose Tissue Inflammation in Young, Lean Male Mice Offspring

**DOI:** 10.1210/en.2016-1314

**Published:** 2016-09-01

**Authors:** Maria Z. Alfaradhi, Laura C. Kusinski, Denise S. Fernandez-Twinn, Lucas C. Pantaleão, Sarah K. Carr, David Ferland-McCollough, Giles S. H. Yeo, Martin Bushell, Susan E. Ozanne

**Affiliations:** Metabolic Research Laboratories and Medical Research Council (M.Z.A., L.C.K., D.S.F.-T., L.C.P., S.K.C., G.S.H.Y., S.E.O.), Metabolic Diseases Unit, University of Cambridge, Cambridge, CB2 0QQ, UK; and Medical Research Council Toxicology Unit (D.F.-M.), University of Leicester, Leicester, LE1 9HN, UK

## Abstract

Obesity during pregnancy has a long-term effect on the health of the offspring including risk of developing the metabolic syndrome. Using a mouse model of maternal diet-induced obesity, we employed a genome-wide approach to investigate the microRNA (miRNA) and miRNA transcription profile in adipose tissue to understand mechanisms through which this occurs. Male offspring of diet-induced obese mothers, fed a control diet from weaning, showed no differences in body weight or adiposity at 8 weeks of age. However, offspring from the obese dams had up-regulated cytokine (*Tnf*α; *P* < .05) and chemokine (*Ccl2* and *Ccl7*; *P* < .05) signaling in their adipose tissue. This was accompanied by reduced expression of miR-706, which we showed can directly regulate translation of the inflammatory proteins IL-33 (41% up-regulated; *P* < .05) and calcium/calmodulin-dependent protein kinase 1D (30% up-regulated; *P* < .01). We conclude that exposure to obesity during development primes an inflammatory environment in adipose tissue that is independent of offspring adiposity. Programming of adipose tissue miRNAs that regulate expression of inflammatory signaling molecules may be a contributing mechanism.

Over recent years, there has been an alarming rise in the prevalence of obesity in Western populations including women of childbearing age (1, 2). This is of concern as obesity during pregnancy is associated with detrimental outcomes for both mother and child, including increased risk of preeclampsia and complicated delivery (3). As well as these immediate detrimental consequences, it is also thought that obesity during pregnancy has long-term effects on the health of the offspring as a consequence of “developmental programming.” This is based on the notion that conditions presented during critical windows of development lead to permanent programmed alterations in physiological systems and consequently organ function and whole-body metabolism (4). Although initial studies in this field focused on fetal undernutrition/low birth weight and increased risk of heart disease (5) and glucose intolerance/type 2 diabetes (6), in light of the current obesity epidemic there is now a growing focus on the detrimental effects of overnutrition during pregnancy (reviewed in Ref. 7).

In humans, the strongest evidence for the programming effects on offspring health by obesity during pregnancy has come from the study of siblings who were born before and after their mother had bariatric surgery to reduce her adiposity. These have shown that siblings born postsurgery are leaner, more insulin sensitive and have lower blood pressure than their sibling born before maternal surgery and hence when the mother was obese (8–10). These observational studies in humans suggesting that an obesogenic in utero environment has programming effects on the offspring have been supported by studies in animal models. Numerous studies in animals have shown that there is a causal relationship between maternal overnutrition during pregnancy and offspring obesity, insulin resistance, fatty liver, and metabolic syndrome (11–13). We and others have shown that these programmed effects on metabolism are associated with mitochondrial dysfunction and altered expression of insulin signaling proteins in skeletal muscle and liver (14–16).

Adipose tissue is known to play an important role in the development of metabolic syndrome. It plays a pivotal role in maintaining metabolic homeostasis through its ability to store and release lipids, take up glucose in an insulin-responsive manner, and through the production of adipokines (17, 18). It has been demonstrated that adipose tissue is vulnerable to the effects of a suboptimal early environment and therefore can contribute to the programming of whole-body metabolic dysfunction. Molecular analyses that have adopted a candidate approach have demonstrated that diet-induced obesity during pregnancy can lead to programmed effects on mRNA, micro RNA (miRNA), and protein expression in adipose tissue. These include studies that have shown significant reduction in expression of glucose transporter type 4 in adipose tissue, which can selectively cause insulin resistance (19), and our own studies, which demonstrate up-regulation of miR-126 that can directly influence insulin receptor substrate-1 protein expression (20).

The aim of the current study was to take an unbiased approach to detect programmed changes in mRNA and miRNA levels in adipose tissue that could contribute to offspring metabolic dysfunction in later life. To achieve this we carried out mRNA and miRNA array analyses on adipose tissue from the male offspring of obese mouse dams. We studied offspring in young adult life when they were lean to enable us to identify drivers of potential future metabolic dysfunction rather than changes that were a consequence of alterations in current adiposity.

## Materials and Methods

### Animal model

All studies were approved by the local Ethics Committee and were conducted according to the Home Office Animals (Scientific Procedures) Act 1986. The model of maternal diet-induced obesity used here has been described previously (20). Briefly, female C57BL/6J mice were fed ad libitum a standard control chow diet (2.56 kcal/g; fat, 7.42% [kcal]; RM1, Special Diet Services) or a highly palatable energy-rich obesogenic diet (6.79 kcal/g; fat, 45% [kcal]; 45% AFE Fat, Special Diet Services) and sweetened condensed milk (55% simple sugar, 8% fat, 8% protein [wt/wt]; Nestle, fortified with mineral and vitamin mix AIN93G) from 4 weeks of age. After 6 weeks on control or obesogenic diets, dams (n = 11 per group) were mated with chow-fed males for first pregnancy. The dams were allowed to litter, and the first litter was culled after weaning. This first pregnancy ensured the mice were proven breeders. After a week, mice were mated for a second pregnancy, and day 1 of pregnancy was defined by the appearance of a plug. Dams were maintained on their respective experimental diets throughout both pregnancy and lactation. Litter sizes were standardized to 3 males and 3 females on postnatal day 3. Maternal body weight was recorded on a weekly basis until weaning, at which time domain nuclear magnetic resonance was performed before dams were culled. All offspring groups were weaned onto standard chow (RM1), fed ad libitum, at 21 days of age, and were maintained on this diet for the remainder of the study. At 8 weeks of age, following an overnight fast, offspring were killed by rising CO_2_ concentration. Epididymal adipose tissue was dissected, weighed, snap frozen, and stored at −80°C until use. Only male offspring were assessed in this study in order to control for metabolic differences which could be influenced by the sex hormones.

### RNA extraction for microarray analyses

Each adipose tissue sample used for the microarrays consisted of grounded, pooled adipose tissue from 2 litters (each litter had 3 male mice, therefore each microarray sample was a pool of a total of 6 mice). RNA was extracted from 50 mg of pooled adipose tissue using a column based *mir*Vana isolation kit according to the manufacturer's protocol. For quantitative PCR analysis (qPCR) analysis for validation of the microarrays, additional RNA from epididymal adipose tissue was extracted from 1 male adipose tissue from individual litters (n = 5 samples per group, where each n was adipose tissue from 1 mouse from a single litter).

### Microarray analysis

All reagents for the microarray analysis were purchased from Agilent Technologies. mRNA expression profiling (n = 4 pooled samples per group) was performed using single-color Agilent SurePrint G3 Mouse GE 8 × 60K Microarrays (design ID, 028005).

Data analysis was performed using GeneSpring GX 12.5 (Agilent). Intensities were normalized to the 75th percentile, and baseline data transformation was performed to the median of the control samples. A *P* < .05 was applied with a 2-fold change cut off. Pathway analysis was then carried out using Ingenuity Pathway Analysis (IPA) software. The pathway analysis identified canonical pathways from the IPA library that were most significant to the dataset.

### microRNA array analysis

miRNA expression profiling (n = 5 pooled samples per group) was performed using Agilent SurePrint Mouse microRNA Microarray slides (design ID, 046065). The data were extracted using the Agilent Feature Extraction software version 10.7.3.1 and analyzed using GeneSpring GX software. Intensities were normalized to the 90th percentile using the percentile shift method. Fold changes were generated for samples expressed above threshold levels and analyzed using unpaired Student's *t* test for significance (*P* < .05).

Putative targets of miR-706 and miR-3472 were scanned using the microRNA database miRWalk (21). Predicted 3′-untranslated region (UTR) target binding sites for miR-706 were compared across 4 target prediction databases: miRanda, miRDB, miRWalk, and TargetScan. A total of 168 targets were commonly identified across databases. A similar comparison was not available for miR-3472, due to the lack of characterization of this relatively newly identified miRNA. Putative miR-706 targets were then crosschecked with the transcript list generated from the microarray to determine whether putative miR-706 targets were dys-regulated in maternal obese (Mat-Ob) offspring adipose tissue.

### Quantitative PCR analysis

The top 5 up-regulated and 5 down-regulated transcripts, identified by the microarray analysis, were selected for validation by qPCR using the same samples of pooled adipose tissue that were included in the array plus additional adipose tissue samples from a single male mouse from different litters (n = 8–11 per group) as previously described (22). For primer sequences, see Supplemental Table 1. mRNA expression was normalized to the geomean of the housekeeping genes β-*actin*, *Gapdh*, and *Ppia*, the expressions of which did not differ between experimental groups.

miRNAs identified as significantly different between groups were validated by TaqMan miRNA assay (Applied Biosystems) for the miRNAs which had TaqMan assays available: mmu-let-7c-1-3p (assay ID, 002479), mmu-mir-706 (assay ID, 001641), and mmu-mir-3472 (assay ID, 241380_mat). miRNA expression was normalized to the geomean of the housekeepers *rnu6b_2*, *rnu5a*, *rnu1a*, *snord25*, and *scarna17* (QIAGEN miScript primer control set catalog 218300), the expressions of which did not change between the experimental groups.

### Protein quantification

Putative miRNA targets were validated by Western blotting. Protein was extracted from 50 mg of epididymal adipose tissue. Protein lysates were standardized to a concentration of 1 mg/mL and 20-μL total protein was resolved using SDS-PAGE (10%). Calcium/calmodulin-dependent protein kinase 1D (CAMK1D) primary antibody was diluted 1:10 000 in 5% BSA, 1× Tris-buffered saline, 0.1% Tween 20 (ab172618; Abcam). IL-33 primary antibody was diluted 1:500 in 1% nonfat dehydrated milk, 1× Tris-buffered saline, 0.1% Tween 20 (sc-98660; Santa Cruz Biotechnology). Horseradish peroxidase-conjugated secondary antibodies (antirabbit/mouse/goat antibody; Jackson ImmunoResearch) were used. Protein expression was quantified by densitometry using AlphaEase software (AlphaInnotech).

### Adipose tissue histology

Epididymal adipose tissue was fixed and paraffin embedded, and 2 5-μm sections per sample n = 5–6 per group were mounted onto a positively charged slide. For adipocyte size and number analysis, tissue was stained with hematoxylin and eosin following a standard protocol. Automated analysis was performed using cell P software. Macrophage and T-cell infiltration in the adipose sections was assessed using an antibody specific to macrophages (F4/80) and T cells (CD3), following a standard protocol. In brief, slides were rehydrated, treated with proteinase K, and incubated with 0.3% hydrogen peroxidase. Samples were treated with blocking solution and then incubated with primary antibody F4/80 (Bio-Rad) or CD3 (Abcam) at 4°C overnight. Secondary antirat IgG antibody (Jackson ImmunoResearch) for F4/80 and antirabbit IgG antibody (Dako) for CD3 was used. Macrophage number and T-cell number were counted blindly in 10 fields of view per section at ×20 magnification, using ImageJ analysis software.

### Luciferase assay

Luciferase reporter constructs were generated by subcloning either the IL-33-3′-UTR target sequence of miR-706 or a mutated IL-33-3′-UTR target sequence downstream of the luciferase gene in a pGL3-control vector (Promega). HeLa cells were seeded in 24-well plates at a density of 5 × 10^4^ cells/well. After 24 hours, cells were transfected with 100 ng of the firefly-luciferase pGL3 construct (containing either the wild-type or the mutated target sequence), plus 10 ng of *Renilla*-luciferase pRL-TK (Promega) used for normalization of the firefly luciferase activity using Lipofectamine 2000 transfection reagent (Invitrogen). Cells were cotransfected with 10nM mmu-miR-706 mimic or a negative control RNA (Life Technologies). Cells were harvested and lysed after 24 hours of transfection and both firefly and *Renilla*-luciferase activity was measured using a DualGlo Luciferase Assay System (Promega). Luciferase activity in lysates of cells transfected with the mmu-miR-706 mimic was normalized by the signal acquired in the respective negative control transfection.

### Statistical analysis

In each case, n refers to the number of adipose tissue samples per group. For microarray data, each n was a pool of 6 mice from 2 litters. For all other data, n refers to the number of mice from different litters. For normally distributed data, results are shown as mean ± SEM, and data were analyzed using unpaired Student's *t* tests. Data that did not pass the normality tests are shown as median with interquartile range (IQR) and were analyzed by the Mann Whitney *U* test. For all data, *P* < .05 was considered statistically significant.

## Results

### Maternal body weight and composition

Feeding dams an obesogenic diet resulted in significantly increased body weight at the start of pregnancy compared with mice fed a control diet. This was maintained throughout pregnancy and lactation (Table 1), similar to findings previously published (23). Body composition of the dams analyzed at the end of lactation showed that dams fed the obesogenic diet had significantly higher body fat mass, both in absolute terms and as a percentage of body weight. Lean mass in relative terms was reduced but unchanged in absolute terms (Table 2).

**Table 1.** Body Weights of Control and Diet-Induced Obese Dams Throughout Pregnancy and Lactation

Body Weight (g)	Control Dams	Obese Dams
Pregnancy day 2	28.4 ± 0.7	34.1 ± 2.4^a^
Lactation day 2	32.2 ± 0.4	35.0 ± 1.1^a^
Lactation day 21	33.5 ± 0.5	40.2 ± 2.3^b^

n = 11 per group, values presented as mean ± SEM.

a *P* < .05.

b *P* < .01.

**Table 2.** Body Composition of Control and Diet-Induced Obese Dams at the End of Lactation

	Control Dams	Obese Dams
Lean mass (g)	19.6 ± 0.3	19.7 ± 0.3
% Lean mass	72.1 ± 0.6	55.9 ± 2.7^a^
Fat mass (g)	3.1 ± 0.3	12.9 ± 2.0^a^
% Fat mass	11.3 ± 0.7	33.7 ± 3.3^a^

n = 11 per group, values presented as mean ± SEM.

a *P* < .001.

### Offspring whole-body phenotype

**Figure 1.**
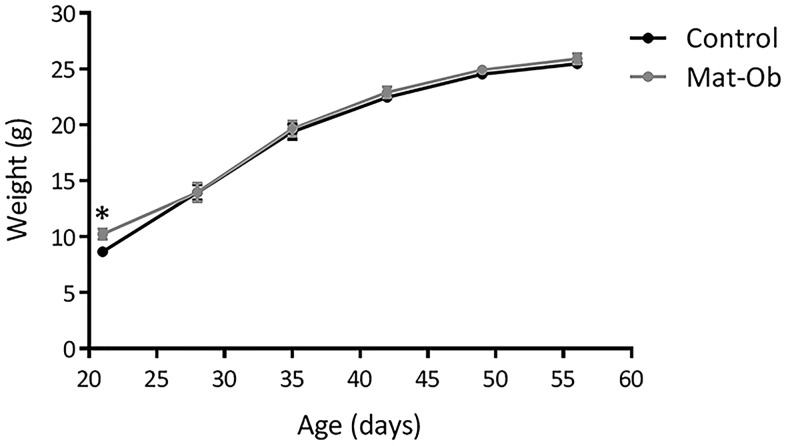
Offspring phenotype at 8 weeks of age. Postweaning body weight trajectory; *, *P* < .05, n = 11 per group.

As expected, based on previous studies, there were no differences in body weight (Figure 1) or adiposity (relative total [epididymal, retroperitoneal, and ip] fat pad weight control, 13.4 ± 1.05-mg/g body weight vs Mat-Ob 13.5 ± 1.06-mg/g body weight) between the groups at 8 weeks. There were also no differences in fasted levels of plasma triglycerides (control, 1.5 mmol/L [IQR, 1.2–2.3] vs Mat-Ob, 1.4 mmol/L [IQR, 1.0–1.6]), cholesterol (control, 3.22 ± 0.11 mmol/L vs Mat-Ob, 3.33 ± 0.09 mmol/L) or glucose (control, 6.5 ± 0.8 mmol/L vs Mat-Ob, 6.2 ± 0.4 mmol/L). This therefore confirmed that any differences that we identified in gene expression profile in adipose tissue were not related to any changes in adiposity or circulating lipid levels.

### Global gene expression identifies up-regulated inflammatory profile

**Figure 2.**
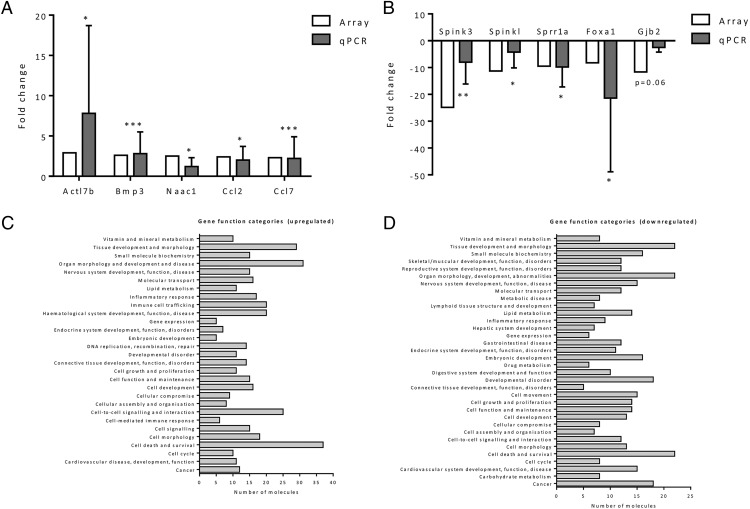
Adipose microarray analysis. qPCR validation of top up-regulated (A) and down-regulated (B) transcripts; gene categories significantly enriched with up-regulated (C) and down-regulated (D) transcripts. qPCR expression was normalized to the geometric mean of the housekeepers β-*actin*, *gapdh*, and *ppia*. *, *P* < .05; **, *P* < .01; ***, *P* < .001 determined by one-tailed Student's *t* test, n = 10 for control, n = 11 for Mat-Ob.

Microarray analysis was performed to detect global changes and patterns in mRNA content in response to the exposure to maternal obesity in early life. The microarray contained 55 821 probes, representing 39 430 Entrez genes and 16 251 long noncoding RNAs. After the removal of long noncoding RNAs, predicted/unannotated genes, and pseudo/chromosomal genes from the analysis, a total of 14 601 transcripts (37% of those represented on the array) were expressed above threshold levels in offspring adipose tissue at 8 weeks of age. A total of 178 transcripts in offspring adipose tissue were significantly (*P* < .05) differentially expressed by greater than 2-fold after exposure to maternal diet-induced obesity. A total of 122 gene transcripts were significantly up-regulated, and 53 gene transcripts were significantly down-regulated in 8-week-old Mat-Ob offspring epididymal adipose tissue compared with controls (full list of transcripts in Supplemental Tables 2 and 3). Differential expression of 9 of the 10 transcripts (90%) chosen for validation was confirmed by qPCR (Figure 2, A and B).

IPA identified gene function categories and signaling pathways that were significantly populated with transcripts from the microarray analysis. In total, 28 pathways were enriched with up-regulated transcripts (Supplemental Table 4). These included genes involved in inflammatory response, immune cell trafficking, and cell-mediated immune responses (*Il1*β, *Tnf*α, *Ccl2*, and *Ccl7*) (Figure 2C and Supplemental Table 4). Thirty-eight pathways were enriched with down-regulated transcripts. These included genes involved in lipid and carbohydrate metabolism (Figure 2D and Supplemental Table 5).

**Figure 3.**
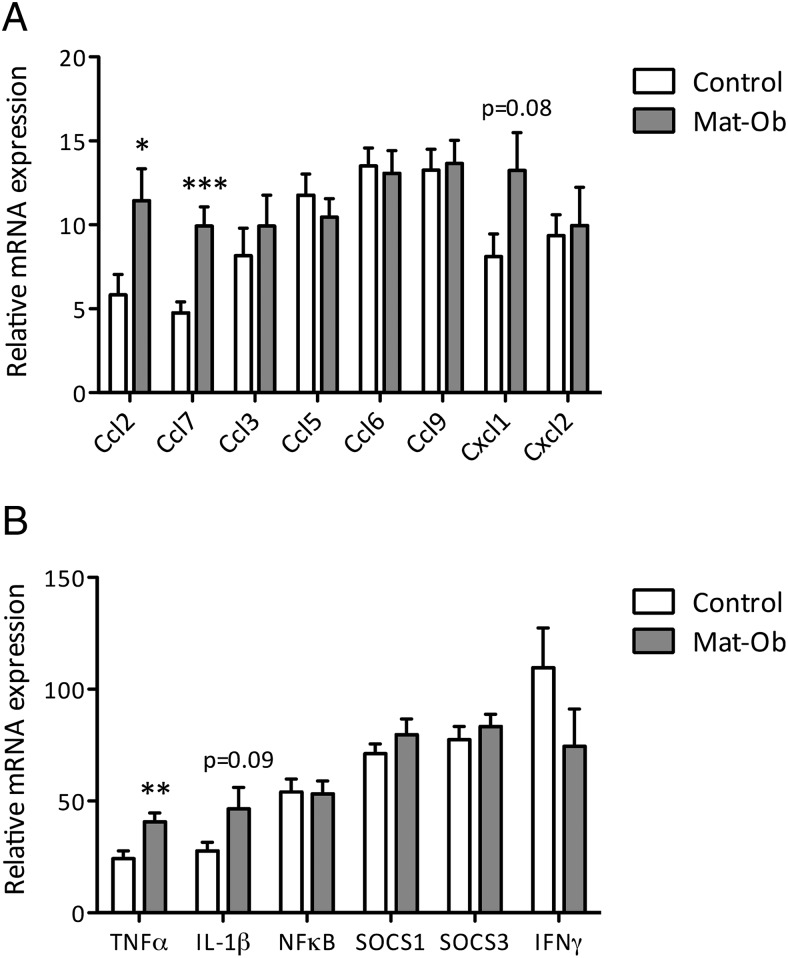
Inflammation in 8-week-old male adipose tissue. A, Chemokine expression. B, Inflammatory transcription factor expression at mRNA level. qPCR expression was normalized to the geomean of the housekeepers β-*actin*, *gapdh*, and *ppia*. *, *P* < .05; **, *P* < .01; ***, *P* < .001 determined by 2-tailed Student's *t* test, n = 10 for control, n = 11 for Mat-Ob.

Because obesity is considered a chronic inflammatory disease, and inflammatory chemokines and transcription factors were identified in the microarray, we further profiled the expression of chemokines and transcription factors involved in inflammatory cell recruitment that have been reported as up-regulated in obese individuals. None of these additional chemokines or inflammatory transcription factors were found to be up-regulated in Mat-Ob offspring just the *Ccl2*, *Ccl7*, and *Tnf*α identified in the microarray (Figure 3, A and B), suggesting these genes are selectively sensitive to programming. In addition, circulating levels and epididymal fat pad expression of leptin were significantly elevated in Mat-Ob offspring at 8 weeks of age (*P* = .016 and *P* = .018, respectively) (Table 3).

**Table 3.** Leptin Expression at 8 Weeks of Age

	Control	Mat-Ob
Serum leptin (pg/mL)	523 ± 122	1035 ± 142^a^
Adipose tissue leptin mRNA expression	4.08 ± 0.6	16.44 ± 4.24^a^

Values presented as mean ± SEM or median (IQR).

a *P* < .05 determined by 2-tailed Student's *t* test, n = 8 per group.

### Hypertrophied adipose tissue

In light of the data described above, we further examined adipose tissue to determine whether up-regulated *leptin*, *Ccl2*, *Ccl7*, and *Tnf*α could result in localized macrophage recruitment/infiltration.

**Figure 4.**
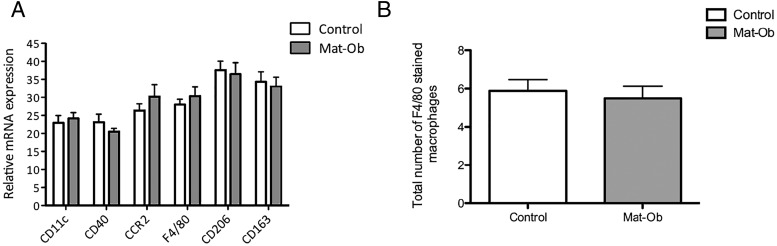
Inflammatory cell markers in 8-week-old male epididymal adipose tissue. A, qPCR expression of classical inflammatory cell markers, normalized to the geomean of the housekeeping genes β-*actin*, *gapdh*, and *ppia* (n = 10 for control, n = 11 for Mat-Ob). B, Average macrophage cell count per field of view (n = 5 for control, n = 6 for Mat-Ob).

**Figure 5.**
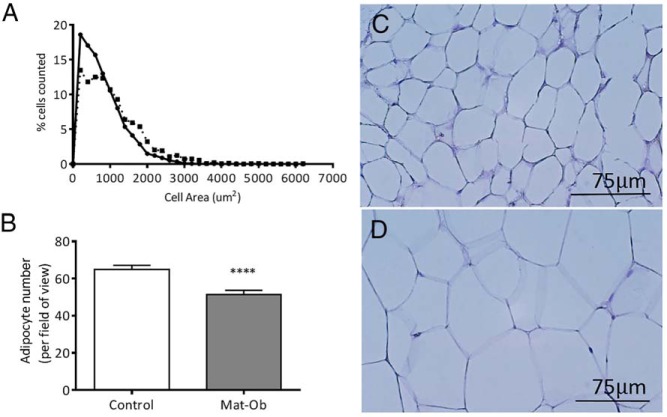
Adipose tissue histology in 8-week-old males. A, Adipocyte cell size analysis. B, Adipocyte cell number. Representative images of hemotoxylin stained adipocytes from control (C) and Mat-Ob (D) groups. Scale bar, 75 μm.

We examined the expression of macrophage markers by qPCR and detected no significant differences in their expression between the 2 groups (Figure 4A). We also stained adipose tissue sections with F4/80 to detect possible subtle changes in macrophage numbers in offspring adipose tissue. However, there was no change in the number of macrophages observed within the Mat-Ob offspring adipose tissue compared with control (Figure 4B). In addition, there was no detectable infiltration of T cells in the adipose tissue (data not shown). Adipose tissue histology showed that adipocytes in Mat-Ob offspring were significantly larger in size (control, 682 μm^2^ [787–829 μm^2^ 95% confidence interval] and Mat-Ob, 898 μm^2^ [1024–1087 μm^2^ 95% confidence interval] values represent median; *P* < .001); hence, there were fewer in number, per field of view, when compared with control offspring (Figure 5, A–D).

### Global microRNA expression

**Figure 6.**
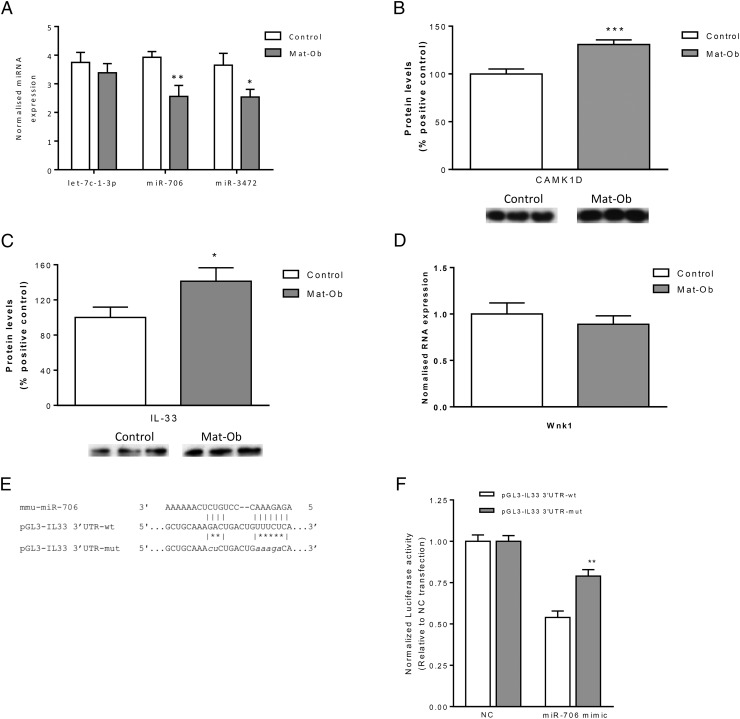
microRNA and putative target validation. A, qPCR validation of selected miRNA from the microarray, normalized to the geomean of the housekeepers rnu6b_2, rnu5a, rnu1a, snord25, and scarna17 (n = 10 and 11 for control and Mat-Ob, respectively). B, Protein levels of CAMK1D determined by Western blotting with representative blots (n = 8 per group). C, Protein levels of IL-33 determined by Western blotting with representative blots (n = 8 per group). D, mRNA levels of *Wnk1* (n = 10 per group). E, Target sequence and mutated target sequence of miR-706 on IL-33 3′-UTR subcloned in a pGL3-enhancer vector (pGL3-IL33 3′-UTR-wt and pGL3-IL-33 3′-UTR-mut, respectively). F, Normalized luciferase activity of the wild-type (pGL3-IL-33 3′-UTR-wt) and mutant (pGL3-IL33 3′-UTR-mut) IL-33 3′-UTR/miR-706 binding site in HeLa cells after negative control (NC) or miR-706 mimic transfection (n = 6 per group); *, *P* < .05; **, *P* < .01; ***, *P* < .001.

miRNA mediated regulation of gene expression at the translation stage may be a potential mechanism underlying developmental programming. We profiled the global expression of miRNAs using a microarray platform. The microarray represented 1247 unique mouse miRNAs, corresponding to miRBase v.17.0. A total of 1090 miRNAs were detected in mouse adipose tissue above background levels, representing 87% expression of those represented. Of these, 4 miRNAs were significantly differentially expressed (*P* < .05) in Mat-Ob compared with controls (Table 4). A significant down-regulation of miR-706 and miR-3472 was confirmed in Mat-Ob offspring; there was no significant difference let-7c-1-3p between the 2 groups (Figure 6A). Of these 2 validated targets, we focused our studies on miR-706, because detailed information on miR-3472 and its targets was not available at the time of analysis. We searched for potential miR-706 targets using the miRWalk miRNA database, as described in Materials and Methods. Our search identified 2 putative targets related to inflammation: CAMK1D and IL-33.

**Table 4.** miRNAs Differentially Regulated in 8-Week-Old Male Offspring

microRNA	*P* Value	Fold Change
let-7c-1-3p	.0026	1.66
miR-706	.0085	−1.42
miR-3472	.0403	−1.53
miR-324-3p	.0406	−1.10

### microRNA inflammation pathway target analysis

Transcript levels of CAMK1D and IL-33 were not up-regulated in the microarray analysis. However, miRNAs are thought to act posttranscriptionally through regulation of translation, so protein expression levels of these 2 genes were determined. CAMK1D levels were significantly (*P* < .001) elevated by 30% in the Mat-Ob offspring (Figure 6B). IL-33 protein levels were also significantly (*P* < .05) increased by 41% in Mat-Ob offspring compared with controls at 8 weeks of age (Figure 6C).

miR-706 lies within intron 1 of its host gene WNK lysine-deficient protein kinase 1 (*Wnk1*). The expression of *Wnk1* did not differ between the 2 experimental groups (Figure 6D), suggesting miR-706 is regulated independently of its host in this model.

We then used a luciferase reporter system to show that there was a direct interaction between miR-706 and its putative target sequence within the 3′-UTR of IL-33. HeLa cells were transfected with luciferase constructs containing the target sequence for miR-706 in the 3′-UTR (Figure 6E). Upon exposure to miR-706 mimetic, cells bearing the IL-33 constructs showed an inhibition in luciferase activity. The specificity of the effect was confirmed by using a mutated version of the binding site within the 3′-UTR (Figure 6F).

## Discussion

In this study, we used a genome-wide approach to profile adipose tissue transcription and miRNA expression in 8-week-old mice offspring after exposure to maternal diet-induced obesity during gestation and lactation. Offspring were fed a control diet from weaning and studied at 8 weeks of age in order to determine potential underlying mechanisms that may influence offspring risk of metabolic disorder independent of differences in total adiposity between the 2 groups. As adipose tissue is considered to be an endocrine organ as well as the primary site of lipid storage in the body, dysregulated gene expression can potentially impact on whole-body metabolism.

This study identified many genes in the microarray which were both up and down-regulated across different pathways. Many of these pathways are interesting such as cell proliferation and development, which could have a significant impact on the metabolic phenotype seen in this model. For this study, inflammation became the main area of focus, because this category had the most up-regulated pathways associated with it. Inflammatory response, immune cell trafficking, and cell-mediated immune responses were all up-regulated in the Mat-Ob group.

Our approach revealed significant alterations in Mat-Ob offspring adipose tissue gene expression, with an up-regulation of genes involved in cytokine signaling, inflammatory response, and development. Obesity has long been recognized as a chronic low-grade inflammatory disease. However, in the current study, we observe a proinflammatory profile in offspring from obese dams that precedes differences in adiposity. Adipose tissue inflammation in obese subjects, both in mice and humans, has been associated with insulin resistance. Studies have demonstrated an increased inflammatory profile in rodent models before the onset of impaired insulin resistance. High-fat diet (HFD)-fed mice, for example, showed increased inflammation specifically in adipose tissue after only 3 weeks feeding on such a diet (24). In the current study, this increased inflammation is observed in animals fed a chow diet. TNFα, a proinflammatory cytokine, has been shown to directly mediate insulin resistance in rodent models of obesity (25). Previous studies on fetal adipose tissue from 60% HFD-fed mice dams showed that *TNF*α is increased in fetal adipose tissue as an immediate consequence of maternal high-fat feeding (19). We observed TNFα to be increased in the adipose tissue from offspring of obese dams in adulthood demonstrating that the increase may be programmed and present beyond the period of exposure to the obesogenic environment. Therefore, these findings suggest that programming of an early inflammatory adipose tissue phenotype may be a mechanism that underlies the development of metabolic disorders as a consequence of maternal obesity.

Chemokines play an important role during inflammation, because their purpose is to regulate cell trafficking. Both chemokine C-C-motif chemokine ligand 2 (CCL2) and CCL7 expression was significantly higher in adipose tissue from the offspring of obese dams. CCL2, which is also known as monocyte chemoattractant protein-1, acts through promoting infiltration of macrophages and recruiting monocytes and other inflammatory cell markers (26, 27). CCL7, also known as monocyte chemotactic protein-3, plays a role in chemotaxis of monocytes (28). These 2 chemokines were increased as an immediate consequence of maternal high-fat feeding (29). Our findings demonstrate that their increase may be programmed in adipose tissue as a consequence of maternal diet-induced obesity and is retained after the period of exposure. In humans, CCL2 and CCL7 have been shown to be important in orchestrating the migration of many immune cells such as T-cells and natural killer cells to the area of inflammation (30, 31). In the current study, we were not able to show alterations in macrophage infiltration in adipose tissue at 8 weeks of age. This is consistent with this time point being an early stage of the inflammatory process, which is likely to increase with age and as the animal becomes obese.

An important aspect of our findings was that the adipose tissue inflammation occurred before any difference in adiposity. Although adiposity was not different between offspring groups, Mat-Ob fat depots contained a higher proportion of larger adipocytes than the controls. In addition, we demonstrated that the offspring from Mat-Ob had higher leptin mRNA levels in their adipose tissue and increased serum leptin levels. This is consistent with previous studies suggesting that larger adipocytes express higher levels of leptin (32, 33). This hyperleptinemia may significantly impact on the triglyceride storage capacity of the mature adipocytes in the Mat-Ob group, which may further contribute to the inflammatory state. A number of studies suggest that the expansion of visceral fat stores, as seen here, also leads to the increasing levels of reactive oxygen species that can stimulate production of inflammatory cytokines.

Our genome-wide miRNA profiling revealed differential expression of only a very small number of miRNAs represented on the array (0.32%). It is not clear if the low number identified is a reflection of low sensitivity of the methodology for miRNA analysis or a reflection that very few miRNAs are differentially expressed in adipose tissue from the offspring of obese dams compared with controls. There are very few genome-wide miRNA-profiling studies in maternal programming models; however, the few studies performed to date using the same methodology have also demonstrated a low number of differentially expressed miRNAs. The hepatic expression of approximately 5.7% of 579 miRNAs measured were altered in the offspring of HFD-fed dams at 15 weeks of age and the majority (∼4%) were found to be down-regulated (34). Many of the miRNAs identified had common targets, suggesting that regulation of specific genes might be coordinated by a small number of miRNAs. In addition, in an ovine model of maternal obesity, fetal skeletal muscle expressed 155 of the tested miRNAs, of which only 3 were differentially regulated in response to maternal obesity exposure (35). The advancement in technology and the current availability of deep sequencing technology to assess miRNA expression will help address this question as to whether the small number of programmed miRNAs identified reflects limitations in previous technology or biology.

Of the programmed miRNAs identified in this study, we demonstrated that miR-706 had biological actions that were consistent with a programmed inflammatory phenotype. Because miRNAs generally function to repress protein expression, we expected an increased translation of their target mRNAs. This was indeed the case for 2 putative targets of miR-706: CAMK1D and IL-33, both of which are involved in inflammatory signaling and were found to be significantly elevated at the protein level in adipose tissue from programmed offspring. IL-33 is required for the homeostasis of immune cell regulation, particularly T regulatory cells. IL-33-deficient mice show significant reduction in T regulatory cells in visceral adipose tissue, a trait which suggests that IL-33 plays an important role in maintaining cell homeostasis (36). Elevation of IL-33, as seen here, has been shown to induce production of Th2 cytokines such as IL-5 and IL-13, decrease lipid storage, and alter expression of genes associated with lipid metabolism (37). This process may be an attempt by the adipose tissue to try and restrict lipid accumulation and, thus, restoring functionality in the tissue. CAMK1D is currently not well characterized in terms of biological function, however, as well as being involved in inflammatory signaling, evidence from the liver suggests that it is important for glucose metabolism (38). The impact of increased of CAMK1D and IL-33 expression on adipocytes warrants further investigation. The data from the current study suggest a mechanistic role for miR-706 in contributing to the inflammation in adipose tissue programmed by maternal obesity. The complete spectrum of targets of miR-706 in adipose tissue and the phenotype consequences of its dysregulation are unknown. Because miRNAs often act through regulation of translation, this would require detailed proteomic analysis.

In conclusion, our genome-wide approach has identified potential mechanisms underlying the programming of metabolic disease through maternal obesity. We provide evidence that in response to external stimuli, such as maternal diet-induced obesity, the adipose tissue transcriptome of the resulting offspring, when fed a control diet, is dynamically regulated. We demonstrated an elevation in inflammatory signaling at 8 weeks of age, independent of offspring adiposity that was accompanied by a larger adipocyte cell size. miRNAs, although less widely affected, also contributed to the posttranscriptional regulation of expression of inflammatory signaling molecules. We postulate that this inflammation may contribute to the underlying mechanisms resulting in metabolic dysfunction as a consequence of maternal obesity.

## Appendix

**Table 5.** Antibody Table

Peptide/Protein Target	Antigen Sequence (if Known)	Name of Antibody	Manufacturer, Catalog Number, and/or Name of Individual Providing the Antibody	Species Raised in; Monoclonal or Polyclonal	Dilution Used
CAMK1D		Anti-CAMK1D	Abcam, ab172618	Rabbit; monoclonal [EPR3536 (2)]	1:10 000
IL-33	MRPRMKYSNSKISPAKFSSTAGEALVPPCKIRRSQQKTKEFCHVYCMRLRSGLTIRKETSYFRKEPTKRYSLKSGTKHEENFSAYPRDSRKRSLLGSIQAFAASVDTLSIQGTSLLTQSPASLSTYNDQSVSFVLENGCYVINVDDSGKDQEQDQVLLRYYESPCPASQSGDGVDGKKLMVNMSPIKDTDIWLHANDKDYSVELQRGDVSPPEQAFFVLHKKSSDFVSFECKNLPGTYIGVKDNQLALVEEKDESCNNIMFKLSK	IL-33	Santa Cruz Biotechnology, sc-98660	Rabbit; polyclonal (M-226)	1:500
CD3	KAKAKPVTRGAGA	Anti-CD3 antibody [SP7]	Abcam, ab16669	Rabbit; monoclonal [SP7]	1:1000 for WB 1:100 for IHC
F4/80		F4/80 antibody	Bio-Rad, MCA497G	Rat; monoclonal	1:150
